# Mean Platelet Volume-To-Lymphocyte Ratio Predicts Poor Functional Outcomes Among Ischemic Stroke Patients Treated With Intravenous Thrombolysis

**DOI:** 10.3389/fneur.2019.01274

**Published:** 2019-12-10

**Authors:** Si-Yan Chen, Yuan-Shao Lin, Yi-Fan Cheng, Hong Wang, Xiao-Ting Niu, Wan-Li Zhang

**Affiliations:** Department of Neurology, The First Affiliated Hospital of Wenzhou Medical University, Wenzhou, China

**Keywords:** ischemic stroke, mean platelet volume, MPVLR, thrombolysis, outcomes

## Abstract

**Background and Purpose:** According to previous studies, the mean platelet volume-to-lymphocyte ratio (MPVLR) represents a novel marker of a poor short-term prognosis in patients with a myocardial infarction who underwent primary percutaneous coronary intervention. We aimed to evaluate the association between MPVLR and clinical outcomes of patients with acute ischemic stroke who were treated with intravenous thrombolysis.

**Methods:** Two hundred forty-one patients with ischemic stroke receiving intravenous thrombolysis were prospectively enrolled in this study. Blood samples for MPVLR were obtained at admission and at 18–24 h after treatment with intravenous thrombolysis. A poor functional outcome was defined as a modified Rankin scale score of 3–6 at 3 months after stroke.

**Results:** At admission, the area under the curve of MPVLR to predict poor functional outcomes at 3 months was 0.613 [95% confidence interval (CI), 0.541–0.686; *P* = 0.003), and the best predictive MPVLR value was 5.8. Patients with an MPVLR ≥5.8 had a 3.141-fold increased risk of a poor outcome at 3 months (95% CI, 1.491–6.615; *P* = 0.003) compared to patients with an MPVLR <5.8. At 18–24 h after treatment with intravenous thrombolysis, the area under the curve of MPVLR to predict a poor outcome at 3 months was 0.697 (95% CI, 0.630–0.765, *P* < 0.001), and the best predictive MPVLR value was 6.9. The inclusion of MPVLR as a continuous (odds ratio, 1.145; 95% CI, 1.044–1.256, *P* = 0.004) and categorical variable (odds ratio, 6.555; 95% CI, 2.986–14.393, *P* < 0.001) was independently associated with poor outcomes at 3 months.

**Conclusions:** Both the values of MPVLR at admission and 18–24 h after intravenous thrombolysis were independently associated with poor functional outcomes. MPVLR may serve as an activity marker for a poor prognosis in patients with acute ischemic stroke receiving intravenous thrombolysis.

## Introduction

Intravenous administration of recombinant tissue plasminogen activator (rt-PA) is recommended as the primary treatment for patients with acute ischemic stroke within 4.5 h from symptom onset (1, 2). However, approximately half of the participants were not independent or died at 3 months, despite the administration of intravenous thrombolytic therapy (3). Since the factors related to the functional outcomes of patients with ischemic stroke who are treated with intravenous alteplase have not been well identified (3, 4), the detection of new risk factors is always important.

Hemocyte variables have been reported to predict the risk and prognosis of ischemic stroke, particularly in patients treated with intravenous thrombolysis (5–8). Platelets play an important role in inducing inflammation and contributing to thrombus formation in cardiovascular disease (9, 10). The mean platelet volume (MPV) is an indicator of platelet function, reflecting platelet size, and activity (11). According to Greisenegger et al. an increased MPV is associated with poor functional outcomes in patients with acute ischemic stroke (12). However, the association between MPV and stroke outcomes remains controversial (13, 14). The pathophysiological pathways induced by cerebral ischemia mainly include the immune response, neuroinflammation, and the accumulation of leukocytes, such as lymphocytes, monocytes, and neutrophils (4, 15). Lower lymphocyte counts are independently associated with an increased probability of a poor functional outcome (7). Recently, the MPV-to-lymphocyte ratio (MPVLR) was considered a novel marker of a poor short-term prognosis in patients with myocardial infarction who underwent a primary percutaneous coronary intervention (16). Based on this information, we were prompted to explore the prognostic value of MPVLR in acute ischemic stroke. Therefore, the objective of our study was to explore whether MPVLR was associated with the functional outcomes of patients with acute ischemic stroke who were treated with intravenous thrombolysis.

## Materials and Methods

### Study Population

We performed a prospective, hospital-based observational study. The study consecutively enrolled patients with ischemic stroke who were treated with intravenous thrombolysis from March 2013 to Oct 2017 in the First Affiliated Hospital of Wenzhou Medical University. Each patient received intravenous alteplase at a dose of 0.9 mg/kg. Notably, 10% of the total dosage was administered as a bolus within 4.5 h of stroke onset, followed by a continuous intravenous infusion of the remaining dose over a period of 1 h. The inclusion criteria for intravenous thrombolysis were as follows: (1) older than 18 years, (2) a diagnosis of acute ischemic stroke by neurologists according to the recommendations from World Health Organization (17), and (3) an onset-to-treatment time within 4.5 h. The exclusion criteria were defined according to the 2013 American Heart Association/American Stroke Association Guidelines (18). The present study was approved by the Ethics Committee of the First Affiliated Hospital of Wenzhou Medical University. All patients or their relatives provided written informed consent and agreed to participate in the study.

### Clinical Protocol and Laboratory Tests

The severity of neurological impairment was assessed using the National Institutes of Health Stroke Scale (NIHSS) at admission and at 24 h after intravenous thrombolysis. Baseline information about age, sex, onset-to-treatment time, systolic, and diastolic blood pressure, medical history, including potential stroke risk factors and ongoing antiplatelet therapy, and electrocardiographs were obtained at admission and in the inpatient department. We collected the following poststroke infection parameters within 48 h: pneumonia, tracheobronchitis, urinary tract infections, and other defined infections. In addition, the blood glucose level was measured in the emergency room at admission. All patients were subjected to a routinely performed computed tomographic scan at admission and a follow-up scan at 24 h after thrombolysis, or any other time when the patient's neurological deterioration was noted. We also collected information about the symptomatic intracerebral hemorrhage according to the Safe Implementation of Thrombolysis in Stroke-Monitoring Study definition: a local or remote type 2 parenchymal hemorrhage on imaging 24 h after thrombolysis or earlier if the computed tomographic scan was performed due to neurological deterioration combined with an increase in the NIHSS score of 4 points from baseline or a stroke resulting in death within 24 h (19).

Data were obtained from blood tests performed at admission and at 18–24 h after treatment with intravenous thrombolysis. A complete blood count was determined using the automated hematology analyzer (XE-2100, Sysmex Company, Japan) within 60 min after blood sample collection. The MPVLR was calculated as the ratio of MPV to lymphocytes, the unit of MPVLR is fl/10^9^/L. The platelet-to-lymphocyte ratio (PLR) was calculated as the platelet count divided by the lymphocyte count.

### Outcome Measures

The main outcomes of all patients were evaluated based on the modified Rankin scale (mRS) score recorded in the outpatient department or via a telephone follow-up interview conducted by a trained neurologist. A poor functional outcome was defined as a mRS score of 3–6 at 3 months after stroke. The second endpoint was early neurological improvement (ENI). ENI was defined as a reduction in the NIHSS score ≥8 from baseline or an NIHSS score that decreased to 0 or 1 at 24 h after treatment (20).

### Statistical Analyses

Statistical analyses were performed with the SPSS 22.0 package for Windows (SPSS Inc., Chicago, USA, RRID:SCR_002865). A *P* < 0.05 was considered statistically significant. Continuous variables are reported as the means with standard deviations or medians with interquartile ranges. Categorical variables are reported as percentages. Statistical analyses were performed using Student's *t*-tests and the Kruskal–Wallis test for continuous data or the chi-square test or Fisher's exact test for categorical data. Receiver operating characteristic (ROC) curves were constructed to assess the predictive value of MPV, lymphocyte, PLR, and MPVLR for poor outcomes at 3 months. The method of an optimal cutoff was applied to transform the MPVLR into a categorical variable. We divided the participants into two groups according to the MPVLR cutoff at admission and compared the differences in baseline characteristics between these groups. We also compared the differences in characteristics between the poor and good outcome group. We employed different multivariate logistic regression models to evaluate the associations between MPV, lymphocyte counts, PLR, MPVLR, and poor functional outcomes at 3 months.

## Results

**Figure 1:**
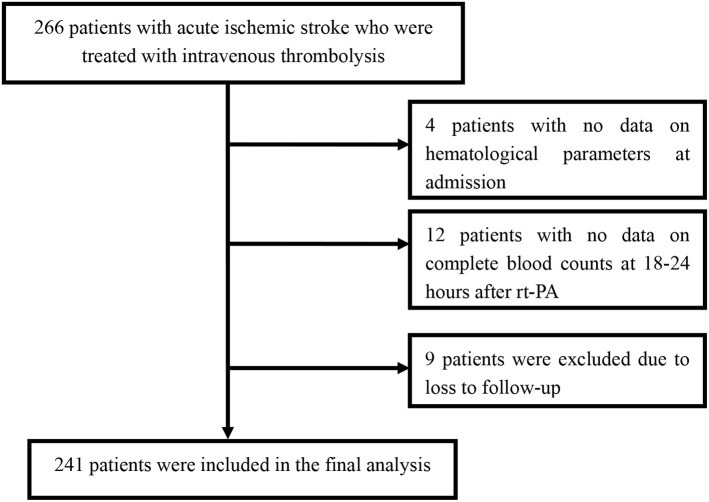
Flow diagram showing the patient selection process.

Among the 266 patients, 12 patients with missing data for the complete blood count at 18–24 h after rt-PA were excluded. Among the remaining patients, four patients with no data on hematological parameters at admission and nine patients who were lost to follow-up were also excluded. Finally, we enrolled 241 patients in this study (Figure 1). A detailed description of the baseline clinical characteristics of the study population is provided in Table 1. The mean age of all patients was 66.5 ± 12.0 years, and 163 patients (67.6%) were male. The median NIHSS score at admission was 9 points. The mean value of the MPVLR for the entire population of participants was 6.7 ± 3.6 fl/10^9^/L at admission and 8.8 ± 4.7 fl/10^9^/L at 18–24 h after rt-PA. Among the 241 patients, 25 (10.4%) died, 29 (12.0%) presented ENI, and 105 (43.6%) presented poor functional outcomes at 3 months.

**Table 1:** Comparison of the baseline characteristics between patients with poor and good functional outcomes.

**Characteristics**	**Total (*n* = 241)**	**Good outcome (*n* = 136)**	**Poor outcome (*n* = 105)**	***P-*value**
Age (year), mean ± SD	66.5 ± 12.0	63.6 ± 12.2	70.2 ± 10.6	0.000
Gender, male, *n* (%)	163 (67.6)	95 (69.9)	68 (64.8)	0.402
Baseline NIHSS score, median (IQR)	9 (5,13)	7 (4,10)	12 (9,17)	0.000
24 h NIHSS score, median (IQR)	7 (4,13)	5 (3,7)	14 (10,18)	0.000
History of smoking, *n* (%)	90 (37.3)	54 (39.7)	36 (34.3)	0.388
Coronary artery disease, *n* (%)	22 (9.1)	9 (6.6)	13 (12.4)	0.123
Hypertension, *n* (%)	166 (68.9)	87 (64.0)	79 (75.2)	0.061
Diabetes, *n* (%)	51 (21.2)	29 (21.3)	22 (21.0)	0.944
Hyperlipidemia, *n* (%)	125 (51.9)	71 (52.2)	54 (51.4)	0.905
Previous stroke/TIA, *n* (%)	31 (12.9)	14 (10.3)	17 (16.2)	0.175
Atrial fibrillation, *n* (%)	79 (32.8)	35 (25.7)	44 (41.9)	0.008
Ongoing antiplatelet therapy, *n* (%)	23 (9.5)	11 (8.1)	12 (11.4)	0.382
Systolic BP (mmHg), mean ± SD	151.0 ± 20.0	149.8 ± 18.8	152.6 ± 21.5	0.277
Diastolic BP (mmHg), mean ± SD	85.8 ± 14.5	86.3 ± 13.6	85.1 ± 15.7	0.510
Glucose level at admission (mmol/l), mean ± SD	7.9 ± 2.8	7.7 ± 2.8	8.0 ± 2.8	0.378
Onset to treatment time, min, mean ± SD	214.0 ± 57.3	209.4 ± 59.7	220.1 ± 53.9	0.153
Hemorrhagic transformation, *n* (%)	56 (23.2)	23 (16.9)	33 (31.4)	0.008
SICH, *n* (%)	14 (5.8)	2 (1.5)	12 (11.4)	0.001
Poststroke infection, *n* (%)	65 (27.0)	17 (12.5)	48 (45.7)	0.000
**Cell count at admission**				
WBC, × 10^9^/L, mean ± SD	8.4 ± 2.8	8.3 ± 2.9	8.4 ± 2.7	0.933
Lymphocytes, × 10^9^/L, mean ± SD	1.7 ± 0.8	1.8 ± 0.8	1.6 ± 0.8	0.022
MPV, fl, mean ± SD	9.3 ± 1.4	9.2 ± 1.4	9.5 ± 1.5	0.105
PLT, × 10^9^/L, mean ± SD	207.1 ± 70.0	216.0 ± 76.5	195.5 ± 59.0	0.024
MPVLR, fl/10^9^/L, mean ± SD	6.7 ± 3.6	6.0 ± 3.1	7.5 ± 4.1	0.002
PLR, mean ± SD	140.1 ± 66.5	134.9 ± 60.6	146.7 ± 73.3	0.173
**Cell count at 18–24 H after rt-PA**				
WBC, × 10^9^/L, mean ± SD	8.7 ± 3.0	7.9 ± 2.2	9.8 ± 3.5	0.000
Lymphocytes, × 10^9^/L, mean ± SD	1.5 ± 0.6	1.6 ± 0.5	1.2 ± 0.5	0.000
MPV, fl, mean ± SD	10.6 ± 1.4	10.7 ± 1.3	10.6 ± 1.5	0.935
PLT, × 10^9^/L, mean ± SD	208.8 ± 65.2	216.7 ± 68.1	198.6 ± 59.9	0.032
MPVLR, fl/10^9^/L, mean ± SD	8.8 ± 4.7	7.5 ± 3.3	10.5 ± 5.6	0.000
PLR, mean ± SD	165.4 ± 85.4	148.4 ± 78.9	187.5 ± 88.6	0.000

*NIHSS, National Institute of Health Stroke Scale; TIA, transient ischemic attack; BP, blood pressure; rt-PA, recombinant tissue plasminogen activator; SICH, symptomatic intracerebral hemorrhage; WBC, white blood cell; MPV, mean platelet volume; PLT, platelet; MPVLR, mean platelet volume-to-lymphocyte ratio; PLR, platelet-to-lymphocyte ratio; SD, standard deviation; IQR, interquartile range*.

An ROC curve analysis was performed to assess the predictive values of MPV, lymphocyte counts, PLR, and MPVLR for poor outcomes at 3 months (Table 2). At admission, the area under the curve (AUC) of MPVLR was 0.613 [95% confidence interval (CI), 0.541–0.686; *P* = 0.003), and the best predictive MPVLR value was 5.8. Furthermore, the predictive value of MPVLR for functional outcomes was better than PLR. Accordingly, at 18–24 h after rt-PA infusion, the AUC of MPVLR to predict a poor outcome at 3 months was 0.697 (95% CI, 0.630–0.765, *P* < 0.001), and the best predictive MPVLR value was 6.9.

**Table 2:** Receiver operating characteristic curves identifying the predictive value of PLR and MPVLR for poor outcomes at 3 months.

	**AUC (95% CI)**	***P*-value**
**Cell count at admission**		
PLR	0.538 (0.464–0.611)	0.318
MPVLR	0.613 (0.541–0.686)	0.003
MPV	0.553 (0.479–0.627)	0.160
Lymphocyte	0.400 (0.327–0.472)	0.008
**Cell count at 18–24 H after rt-PA**		
PLR	0.651 (0.580–0.721)	0.000
MPVLR	0.697 (0.630–0.765)	0.000
MPV	0.504 (0.429–0.579)	0.908
Lymphocyte	0.292 (0.225–0.358)	0.000

*PLR, platelet-to-lymphocyte ratio; MPVLR, mean platelet volume-to-lymphocyte ratio; MPV, mean platelet volume; AUC, area under the curve; CI, confidence interval; rt-PA, recombinant tissue plasminogen activator*.

The baseline and clinical characteristics of the study cohort stratified according to MPVLR cutoff at admission are listed in Table 3. Patients presenting with an MPVLR ≥5.8 were older, had a higher NIHSS score at 24 h after intravenous thrombolysis, and longer onset to treatment time than patients with an MPVLR <5.8. Otherwise, ENI and hyperlipidemia were more prevalent in patients with an MPVLR <5.8. Patients with an MPVLR ≥5.8 more frequently presented with a history of previous stroke/transient ischemic attack (TIA) and atrial fibrillation than patients with an MPVLR <5.8.

**Table 3:** Comparison of the baseline characteristics between subgroups based on the MPVLR cutoff at admission.

**Characteristics**	**MPVLR ≥5.8 (*n* = 122)**	**MPVLR <5.8 (*n* = 119)**	***P*-value**
Age (year), mean ± SD	69.1 ± 10.8	63.8 ± 12.5	0.000
Gender, male, *n* (%)	87 (71.3)	76 (63.9)	0.217
Baseline NIHSS score, median (IQR)	9 (5.5, 13)	8 (5,13)	0.497
24 h NIHSS score, median (IQR)	8 (4, 14.5)	5 (3,13)	0.026
History of smoking, *n* (%)	46 (37.7)	44 (37)	0.907
Coronary artery disease, *n* (%)	12 (9.8)	10 (8.4)	0.699
Hypertension, *n* (%)	81 (66.4)	85 (71.4)	0.399
Diabetes, *n* (%)	30 (24.6)	21 (17.6)	0.187
Hyperlipidemia, *n* (%)	54 (44.3)	71 (59.7)	0.017
Previous stroke/TIA, *n* (%)	21 (17.2)	10 (8.4)	0.041
Atrial fibrillation, *n* (%)	49 (40.2)	30 (25.2)	0.013
Ongoing antiplatelet therapy, *n* (%)	12 (9.8)	11 (9.2)	0.876
Systolic BP (mmHg), mean ± SD	149.8 ± 20.4	152.3 ± 19.6	0.331
Diastolic BP (mmHg), mean ± SD	85.2 ± 15.1	86.4 ± 13.9	0.547
Glucose level at admission (mmol/L), mean ± SD	8.0 ± 2.8	7.7 ± 2.8	0.341
Onset-to-treatment time, min, mean ± SD	228.4 ± 53.6	199.3 ± 57.5	0.000
Hemorrhagic transformation, *n* (%)	31 (25.4)	25 (21.0)	0.419
SICH, *n* (%)	8 (6.6)	6 (5.0)	0.615
Poststroke infection, *n* (%)	39 (32.0)	26 (21.8)	0.077
**Cell count at admission**			
WBC, × 10^9^/L, mean ± SD	8.5 ± 3.2	8.2 ± 2.3	0.306
Lymphocytes, × 10^9^/L, mean ± SD	1.2 ± 0.4	2.3 ± 0.8	0.000
MPV, fl, mean ± SD	9.9 ± 1.3	8.8 ± 1.4	0.000
PLT, × 10^9^/L, mean ± SD	185.0 ± 52.2	229.6 ± 78.5	0.000
MPVLR, fl/10^9^/L, mean ± SD	9.1 ± 3.6	4.2 ± 1.1	0.000
PLR, mean ± SD	168.5 ± 70.4	110.9 ± 47.2	0.000
**Cell count at 18–24 H after rt-PA**			
WBC, × 10^9^/L, mean ± SD	8.9 ± 3.5	8.5 ± 2.4	0.258
Lymphocytes, × 10^9^/L, mean ± SD	1.2 ± 0.5	1.7 ± 0.6	0.000
MPV, fl, mean ± SD	11.0 ± 1.4	10.3 ± 1.3	0.000
PLT, × 10^9^/L, mean ± SD	190.9 ± 51.7	226.9 ± 72.3	0.000
MPVLR, fl/10^9^/L, mean ± SD	10.7 ± 5.3	6.9 ± 3.0	0.000
PLR, mean ± SD	179.7 ± 88.9	150.9 ± 79.3	0.008

*NIHSS, National Institute of Health Stroke Scale; TIA, transient ischemic attack; BP, blood pressure; rt-PA, recombinant tissue plasminogen activator; SICH, symptomatic intracerebral hemorrhage; WBC, white blood cell; MPV, mean platelet volume; PLT, platelet; MPVLR, mean platelet volume-to-lymphocyte ratio; PLR, platelet-to-lymphocyte ratio; SD, standard deviation; IQR, interquartile range*.

**Figure 2:**
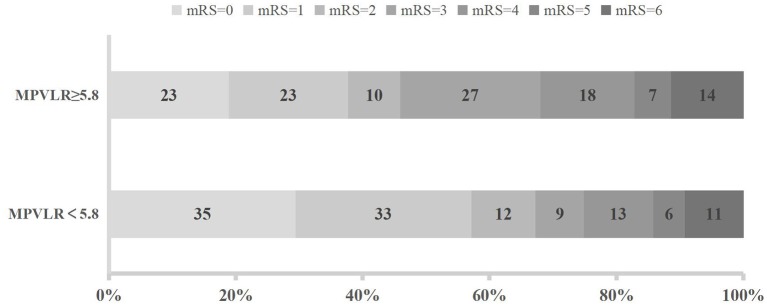
Functional outcomes at 3 months in groups stratified according to the cutoff value of mean platelet volume-to-lymphocyte ratio (MPVLR) at admission.

**Figure 3:**
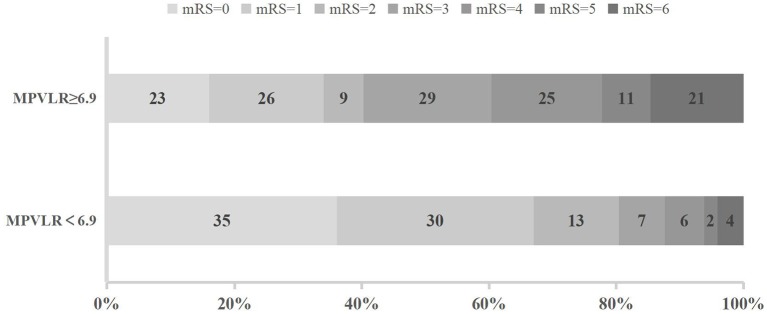
Functional outcomes at 3 months in groups stratified according to the cutoff value of mean platelet volume-to-lymphocyte ratio (MPVLR) at 18–24 h after recombinant tissue plasminogen activator (rt-PA).

The distribution of functional outcomes at 3 months stratified according to the MPVLR cutoff value is shown in Figures 2, 3. Regardless of whether outcomes were measured at admission or at 18–24 h after rt-PA infusion, a poor functional outcome was more common in the higher MPVLR group stratified by cutoff value. However, ENI was less common in the higher MPVLR group stratified by cutoff value (Table 4). The blood tests and the baseline characteristics were compared between patients with poor and good functional outcomes, and the results are shown in Table 1. Patients with poor functional outcomes at 3 months after stroke were older, had higher NIHSS scores, and more frequently suffered from atrial fibrillation and hemorrhagic transformation than patients with good functional outcomes. Before the rt-PA treatment, patients in the poor functional outcome group at admission had lower lymphocyte and platelet counts but higher MPVLR levels than patients in the good functional outcome group. At 18–24 h after rt-PA infusion, white blood cell counts, MPVLR, and PLR were significantly higher in patients with poor functional outcomes than in patients with good functional outcomes. In addition, lower lymphocyte and platelet counts were observed in the poor than in the good functional outcome group.

**Table 4:** Comparison of outcomes between subgroups stratified according to the MPVLR cutoff.

**Outcomes**	**Cell count at admission**		***P*-value**	**Cell count at 18–24 h after rt-PA**		***P-*value**
	**MPVLR ≥5.8 (*n* = 122)**	**MPVLR <5.8 (*n* = 119)**		**MPVLR ≥6.9 (*n* = 144)**	**MPVLR <6.9 (*n* = 97)**	
ENI, *n* (%)	9 (7.4)	20 (16.8)	0.024	11 (7.6)	18 (18.6)	0.011
mRS score, median (IQR)	3 (1,4)	1 (0, 4)	0.009	3 (1,4)	1 (0, 2)	0.000
mRS score of 3–6, *n* (%)	66(54.1)	39 (32.8)	0.001	86 (59.7)	19 (19.6)	0.000

*MPVLR, mean platelet volume-to-lymphocyte ratio; rt-PA, recombinant tissue plasminogen activator; ENI, early neurology improvement; mRS, modified Rankin Scale; IQR, interquartile range*.

Table 5 shows the results of the binary logistic regression analysis of MPV, lymphocyte counts, PLR, MPVLR, and poor outcomes. At admission, the inclusion of the MPVLR level as a dichotomous variable was independently associated with a higher risk of poor outcomes at 3 months, with an adjusted odds ratio (OR) of 2.600 (95% CI, 1.396–4.843; *P* = 0.003) after adjustment for age, sex, and baseline NIHSS score (model 1) and 3.141 (95% CI, 1.491–6.615, *P* = 0.003) after further adjustment for the variables included in model 1 plus variables such as hypertension, coronary artery disease, previous stroke/TIA, atrial fibrillation, onset-to-treatment time, diabetes, hyperlipidemia, poststroke infection, and symptomatic intracerebral hemorrhage that were identified as having *P* < 0.2 in Tables 1, 3 (model 2), respectively. The predictive value of MPVLR as a continuous variable at admission for poor outcomes tended to become significant, regardless of whether it was included in model 1 or 2. At 18–24 h after rt-PA infusion, regardless of whether model 1 or 2 was used, an increased MPVLR level maintained its predictive accuracy as either continuous or categorical variable.

**Table 5:** Multivariate logistic regression analysis of the associations between MPV, lymphocyte, MPVLR, and poor outcome.

		**Multivariate adjusted model (model 1)**	***P*-value**		**Multivariate adjusted model (model 2)**	***P*-value**
	**OR**	**95% CI**		**OR**	**95% CI**	
**Cell count at admission**						
MPV	1.100	0.891–1.358	0.377	1.119	0.886–1.412	0.345
lymphocyte	0.748	0.499–1.120	0.159	0.764	0.481–1.214	0.255
MPVLR	1.092	0.999–1.193	0.053	1.102	0.997–1.217	0.057
MPVLR≥5.8	2.600	1.396–4.843	0.003	3.141	1.491–6.615	0.003
PLR	1.004	0.999–1.009	0.097	1.003	0.998–1.008	0.207
**Cell count at 18–24 H After rt-PA**						
MPV	1.040	0.836–1.295	0.723	1.083	0.848–1.384	0.523
lymphocyte	0.337	0.181–0.626	0.001	0.305	0.148–0.627	0.001
MPVLR	1.134	1.044–1.232	0.003	1.145	1.044–1.256	0.004
MPVLR ≥ 6.9	5.521	2.754–11.067	0.000	6.555	2.986–14.393	0.000
PLR	1.006	1.002–1.009	0.005	1.005	1.001–1.009	0.021

*Model 1, adjusted for age, sex, and National Institutes of Health Stroke Scale score*.

*Model 2, adjusted for the variables included in model 1 plus the variables hypertension, coronary artery disease, previous stroke/TIA, atrial fibrillation, onset-to-treatment time, diabetes, hyperlipidemia, poststroke infection, and symptomatic intracerebral hemorrhage that were identified as having a P < 0.2 in Tables 1, 3*.

*MPV, mean platelet volume; MPVLR, mean platelet volume-to-lymphocyte ratio; PLR, platelet-to-lymphocyte ratio; OR, odds ratio; CI, confidence interval; rt-PA, recombinant tissue plasminogen activator*.

## Discussion

To our knowledge, this study is the first to investigate the prognostic value of the MPVLR in patients with ischemic stroke who were treated with intravenous thrombolysis. When analyzed either pre- or postthrombolysis, the MPVLR was independently associated with increased risks of poor functional outcomes at 3 months. The prethrombolysis value of the MPVLR exhibited a better prediction ability than the PLR. In addition, the best discriminating values of the MPVLR for predicting poor outcomes were determined in this study.

Platelet activation plays an important role in thrombosis and inflammation (21). The size of platelets represents their activity, and larger-sized platelets are functionally more active (22). The MPV is a readily available marker due to the automatic analysis when blood tests were performed. A study including 776 patients with acute ischemic stroke or TIA revealed an adverse effect of a higher MPV son clinical outcome (12). Another study examining patients with ischemic stroke who were receiving intravenous thrombolysis suggested that the MPV was an independent predictor of a poor functional outcome (23), which contradicts the findings from our study. Our study did not observe an association between the MPV and functional outcome. This discrepancy may be due to the use of different designs and outcome measures compared to our study. Furthermore, Ntaios et al. reported that the MPV measured within 24 h after onset was not associated with functional outcomes in patients with ischemic stroke (3).

The inflammatory response after stroke plays an important role in ischemic brain pathobiology (4). The effect of lymphocyte activation on the prognosis of ischemic stroke remains unclear (24). According to the results of some animal experiments, lymphocytes exert a deleterious effect on infarcted brain tissues (5). Another study observed a protective role for regulatory T lymphocytes after experimental brain ischemia (6). Our clinical evidence indicated that lower lymphocyte counts were associated with poor functional outcomes in patients with acute cerebral infarction after rt-PA treatment, consistent with the results reported by Kim et al. (7). Lower lymphocyte counts may be attributed to an increase in inflammation-related lymphocyte apoptosis that predisposes an individual to ischemic stroke.

The PLR predicts clinical outcomes in patients with acute ischemic stroke undergoing thrombectomy (16). However, the platelet size reflects function and activation more accurately than the platelet count (8). In addition, an increased MPVLR has been introduced as a predictor of no-reflow and a poor prognosis in patients with acute myocardial infarction (8, 16). In the present study, an elevated MPVLR was associated with less ENI and worse functional outcomes in patients with ischemic stroke who were treated with intravenous alteplase, suggesting that increased platelet activity and inflammation may be associated with a poor prognosis. The rate of poststroke infection was up to 30%, and the infection might influence hematological parameters, including the lymphocyte count and MPV. We adjusted for poststroke infection in the multivariate regression models, which did not attenuate the risk of poor outcomes associated with MPVLR (25). We also confirmed that the dynamic monitoring of MPVLR at admission and 18–24 h after rt-PA infusion was prospectively associated with poor functional outcomes. In particular, the MPVLR proved to exhibit a better predictive value for poor functional outcomes than the PLR in ROC analyses. The adjusted OR value of MPVLR was also larger than the PLR. We presume that MPVLR may synthetically reflect the degree of thrombosis and inflammation. A potential mechanism is proposed in which an elevated MPVLR may activate platelets by releasing platelet activators from ischemic/necrotic tissue, leading to increased thrombosis (16). However, the present study is not able to confirm a causative role of the MPVLR in the pathophysiology of ischemic stroke. Prospective studies are needed to explore the exact mechanism of this phenomenon.

The current study has certain limitations. First, the MPVLR level and follow-up data were not acquired from 25 patients included in this study. However, no significant differences in the baseline characteristics were observed between the excluded and included patients. Second, the study was only performed at a single center, and thus, the results may not be generalizable to all other patients with ischemic stroke. In addition, the best discriminating values must be further verified in other studies and populations. Furthermore, other platelet markers and inflammatory markers were not evaluated in the study.

## Conclusions

Despite the limitations mentioned above, we report the first study focusing on the predictive value of MPVLR in patients with stroke after intravenous thrombolysis. Moreover, we assessed the MPVLR at admission and at 18–24 h after intravenous thrombolysis, and both values were independently associated with poor functional outcomes. The MPVLR is easy to monitor and therefore might serve as an activity marker for an unfavorable prognosis in patients with acute ischemic stroke receiving intravenous thrombolysis.

## Data Availability Statement

The datasets generated for this study are available on request to the corresponding author.

## Ethics Statement

The studies involving human participants were reviewed and approved by this study was approved by the ethics committee of the First Affiliated Hospital of Wenzhou Medical University. The patients/participants provided their written informed consent to participate in this study.

## Author Contributions

S-YC and W-LZ conceived and designed the study. Y-SL, Y-FC, and HW organized the database. X-TN performed the statistical analysis. S-YC and W-LZ wrote the paper. S-YC, W-LZ, and X-TN reviewed and edited the manuscript. All authors read and approved the manuscript.

### Conflict of Interest

The authors declare that the research was conducted in the absence of any commercial or financial relationships that could be construed as a potential conflict of interest.
